# l-Alpha-glycerylphosphorylcholine can be cytoprotective or cytotoxic in neonatal rat cardiac myocytes: a double-edged sword phenomenon

**DOI:** 10.1007/s11010-019-03580-1

**Published:** 2019-07-06

**Authors:** Eszter Tuboly, Renáta Gáspár, Miguel Olias Ibor, Kamilla Gömöri, Bernadett Kiss, Gerda Strifler, Petra Hartmann, Péter Ferdinandy, Monika Bartekova, Mihály Boros, Anikó Görbe

**Affiliations:** 1 Faculty of Medicine, Institute of Surgical Research, University of Szeged, Szeged, Hungary; 2 Cardiovascular Research Group, Department of Biochemistry, University of Szeged, Szeged, Hungary; 3 Department of Pharmacology and Pharmacotherapy, Semmelweis University, Budapest, Hungary; 4 Pharmahungary Group, Szeged, Hungary; 5 Centre of Experimental Medicine, Institute for Heart Research, Slovak Academy of Sciences, Bratislava, Slovakia; 6 Faculty of Medicine, Institute of Physiology, Comenius University in Bratislava, Bratislava, Slovakia

**Keywords:** Neonatal rat cardiac myocytes, l-Alpha-glycerylphosphorylcholine, Mitochondrial dysfunction, Oxidative stress

## Abstract

l-Alpha-glycerylphosphorylcholine (GPC) is a widely used food supplement. GPC has been shown to exert beneficial effects in several organs; however, the cardiac effects of GPC have yet to be investigated. The aim of the present study was therefore to map out the effects of GPC on cardiac myocytes, with or without ischemia–reperfusion insult. Neonatal rat cardiac myocytes were treated with GPC at 1, 10, 80, and 100 µM concentrations for 15 min, 3 h, or 24 h, respectively. Cell viability by calcein assay and the degree of oxidative stress by DHE (superoxide level) and H_2_DCF (total ROS accumulation) staining were measured. In separate experiments, cardiomyocytes were pre-treated with the optimal concentration of GPC for 3 h and then cells were exposed to 4 h of simulated ischemia followed by 2 h of reperfusion (SI/R). Cell viability was measured at the end of the SI/R protocol. In normoxic conditions, the 15-min and the 3-h GPC treatment did not affect cell viability, total ROS, and superoxide levels. Under SI/R conditions, the 3-h GPC treatment protected the cardiac myocytes from SI/R-induced cell death and did not alter the level of oxidative stress. The 24-h GPC treatment in normoxic conditions resulted in significant cell death and increased oxidative stress at each concentration. Here we provide the first evidence for the cytoprotective effect of short-term GPC treatment. However, long-term administration of GPC may exert cytotoxicity in a wide concentration range in cardiac myocytes. These results may draw attention to a comprehensive cardiac safety protocol for the testing of GPC.

**Electronic supplementary material:**

The online version of this article (10.1007/s11010-019-03580-1) contains supplementary material, which is available to authorized users.

## Introduction

l-Alpha-glycerylphosphorylcholine (choline alphoscerate, GPC) is a natural endogenously produced choline derivative and acetylcholine precursor in the brain which (in the form of a synthetic compound) is widely used as a food supplement [1]. After oral ingestion, it is converted metabolically to phosphatidylcholine, the active form of choline that is able to increase acetylcholine levels in the brain [2–4]. In terms of its use as a food/health supplement, oral GPC intake/supplementation has gained growing attention in both the media and in scientific areas. Since 1998, the Institute of Medicine of the National Academy of Sciences (USA) has recognized choline as an essential nutrient for humans and has made recommendations for the dietary choline intake (Institute of Medicine and National Academy of Sciences 1998). It has been shown that GPC supplements may help substitute for insufficient dietary choline [5]. GPC also contributes to the synthesis of membrane phospho- and glycerolipids, and thus positively influences membrane fluidity [4, 6].

GPC is generally considered a safe and non-toxic compound [4] and it is classified as GRAS (Generally recognized as safe) in the USA (GRN 000419). In Italy and Poland, GPC is a registered drug (Anatomical Therapeutic Chemical Classification System code: n07ax02) marketed under the trade name Gliatilin (produced by Italfarmaco S.p.A., Milan; Italy). It is recommended for use in humans to support brain health and mental capacity. A multicentric, clinical trial confirmed the therapeutic role of alpha-GPC on the cognitive recovery of patients with acute stroke or transient ischemic attack [7]. Along this line, GPC was shown to exert neuroprotective effects in some mental disorders in a series of human clinical studies [8–11].

In addition to its neuroprotective effects, GPC was documented to preserve mitochondrial respiration in liver mitochondria and to reduce hepatic ischemia-induced oxidative stress and inflammation in rodent models of ischemia/reperfusion (I/R) [12, 13]. Protective effects of GPC associated with reduced radical production have been documented also in a relevant rodent model of mesenteric I/R [14]. In view of this, we hypothesized that GPC could prevent oxidative damage following I/R insult in other tissues, including the myocardium.

One concern of GPC is determining the correct dosage, as it is reported that inadequate intake or abnormal metabolism of choline is associated with the increased risk of cardiovascular disease and various cancers [15, 16]. Dose–response experiments were so far restricted to the brain. To the best of our knowledge, the cardiac effects of GPC have yet to be examined.

Therefore, the major objective of the present study was to evaluate the timing and doses of GPC treatment on isolated cultured cardiac myocytes, with a special focus on observing the effects of GPC on the degree of oxidative stress. We aimed to test the hypothesis that GPC could prevent ischemia-induced cell death and oxidative stress in cardiac myocytes subjected to simulated I/R.

## Materials and methods

Our experiments were performed in accordance with the EU directive guidelines for the care and use of laboratory animals, published by the European Union (2010/63/EU). Methods were also reviewed by the National Scientific Ethical Committee on Animal Experimentation (National Competent Authority of Hungary) and were approved by the Animal Welfare Committee of the University of Szeged (I-74-52/2012 MAB).

### Culturing primary neonatal rat cardiac myocytes

Neonatal rat cardiac myocytes (NRCMs) were isolated from newborn Wistar rats using the previously described method [17]. The neonatal rats were sacrificed by cervical dislocation. The hearts were rapidly removed and then placed into a cold (+ 4 °C) phosphate buffered saline (PBS) solution. After the separation of atria, the ventricles were minced with fine forceps and then collected in 0.25% trypsin (Gibco BRL). Tissue fragments were further digested by trypsin for 25 min in a Falcon tube in a 37 °C water bath. Then the cell suspension was centrifuged (450×*g* for 15 min at 4 °C). Afterwards, the cell pellet was re-suspended in culture medium—Dulbecco’s modified Eagle’s medium (DMEM) supplemented with 10% fetal bovine serum (FBS), l-Glutamine, and AB/AM (Sigma). The single cell suspension was pre-plated in six-well plates at 37 °C for 90 min to enrich the culture with cardiac myocytes. The non-adherent myocytes were collected and plated at a density of 10^4^ cells/well onto 96-well plates. The culture medium was changed the day after preparation to a 1% FBS containing proliferation medium. The cells were maintained at 37 °C in a standard CO_2_ incubator (Humidified atmosphere of 5% CO_2_).

### Experimental groups

**Fig. 1:**
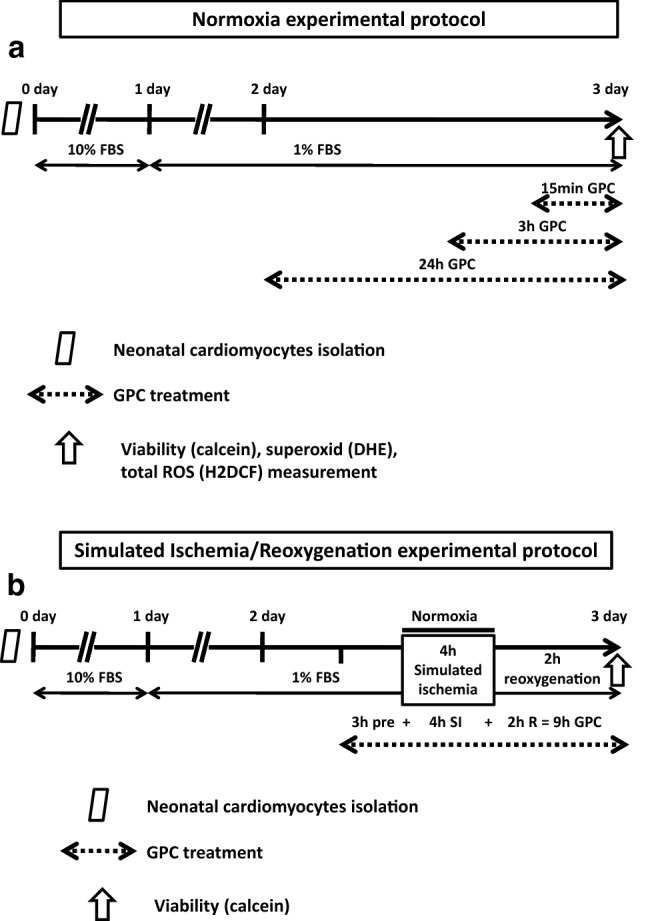
Experimental protocol: after the isolation of the neonatal rat cardiomyocytes (NRCMs) from newborn Wistar rats, the cells were then cultured in a 10% FBS containing medium for 24 h. After the first day, the cells were kept in a 1% FBS containing medium. **a** Glycerophosphorylcholine (GPC) treatment was applied under normoxic conditions. Three series of cells were treated at day 3 with GPC for 15 min, 3 h, or 24 h, respectively, all prior to cell viability measurements. **b** GPC treatment was then applied 3 h prior to 4-h simulated ischemia (SI), and during the 2-h reperfusion period

The concentration-dependent influence of GPC on cell viability and the degree of oxidative stress were tested in 3-day-old primary rat cardiac myocyte cultures exposed to acute (15 min), short-term (3 h), and long-term (24 h) GPC treatment in the 1–100 µM concentration range under normoxic conditions (*n* = 8–16) (Fig. 1a). One group of cells was pre-treated with GPC for 3 h and then subjected to simulated ischemia/reperfusion (SI/R). During the entire protocol, the GPC concentration range (1–100 µM) was sustained (Fig. 1b). In all experiments, GPC was dissolved in physiological saline, while the Vehicle group was treated with physiological saline solution alone in 0.1 v/v%. 2–3 isolation rounds were performed for each experimental series and data of all individual wells were analyzed.

### Simulated ischemia/reperfusion (SI/R)

To simulate ischemic conditions, the culture medium was replaced with a hypoxic solution containing in mM: NaCl 119, KCl 5.4, MgSO_4_ 1.3, NaH_2_PO_4_ 1.2, HEPES 5, MgCl_2_ 0.5, CaCl_2_ 0.9, Na-lactate 20, BSA 0.1% pH 6.4. To induce hypoxia, the cells were then placed into a three-gas incubator, gassed through with a mixture of 95% N_2_ and 5% CO_2_ for 4 h at 37 °C (simulated ischemia). Normoxic control cells were then covered with normoxic solution containing in mM: NaCl 125, KCl 5.4, NaH_2_PO_4_ 1.2, MgCl_2_ 0.5, HEPES 20, MgSO_4_, 1.3, CaCl_2_ 1, glucose 15, taurine 5, creatine-monohydrate 2.5 and BSA 0.1%, pH 7.4 and cells were kept in normoxic incubator. After simulated ischemia or normoxia, the cells were placed to normoxic incubator; hypoxic or normoxic medium was then replaced by culture medium (simulated reperfusion) for 2 h at 37 °C.

### Cell viability assay

Cell viability was assessed by a calcein assay performed at the end of each experimental protocol. In this assay, the cell-permeant calcein-AM dye stains living cells which are then converted to green-fluorescent calcein by intracellular non-specific esterases. The medium was removed, and then the cells were washed with PBS twice and incubated with calcein (1 μM) dissolved in DMSO and further diluted in D-PBS (containing Ca and Mg) for 30 min in a dark chamber. Following this, the calcein solution was replaced with fresh PBS and the fluorescence intensity of each well was measured by a fluorescent plate reader (FluoStar Optima, BMG Labtech) in a well scanning mode (Scan matrix: 10 × 10; scan diameter: 10 mm; bottom optic; no of flashes/scan point: 3; temp: 37 °C; excitation wavelength: 490 nm; emission wavelength: 520 nm).

### Oxidative stress measurements

The level of superoxide (O^2−^) was measured at the end of the experimental protocol by dihydroethidium (DHE) staining, which can visualize the amount of superoxide present in the cells. DHE exhibits blue fluorescence in the cytoplasm and upon oxidation, it intercalates into the DNA and switches to a bright red fluorescence. The fluorescent intensity of each well was detected by a fluorescent plate reader (FluoStar Optima, BMG Labtech) in a well scanning mode (scan matrix: 10 × 10; scan diameter: 10 mm; bottom optic; no of flashes/scan point: 3; temp: 37 °C; excitation wavelength: 530 nm; emission wavelength: 620 nm).

The total reactive oxygen species (ROS) content was measured by cell-permeant 2′-7′-dichlorodihydrofluorescein diacetate (H_2_DCFDA), which is a chemically reduced form of fluorescein and used as an indicator of ROS level in cells. Upon cleavage of the acetate groups by intracellular esterases and oxidation, the non-fluorescent H_2_DCFDA is converted to the highly fluorescent 2′-7′-dichlorofluorescein (DCF). The fluorescent intensity of each well was detected later by a fluorescent plate reader (FluoStar Optima, BMG Labtech) in a well scanning mode (scan matrix: 10 × 10; scan diameter: 10 mm; bottom optic; no of flashes/scan point: 3; temp: 37 °C; excitation wavelength: 480 nm; emission wavelength: 520 nm).

### Statistical analysis

Results are expressed as mean ± SEM. One-way analysis of variance (ANOVA) followed by Dunnett’s multiple comparison post hoc tests were used to analyze differences in mean values of the experimental groups versus vehicle. Differences were considered significant at *p* < 0.05.

## Results

### Acute effect of GPC in isolated NRCMs in normoxic conditions

**Fig. 2:**
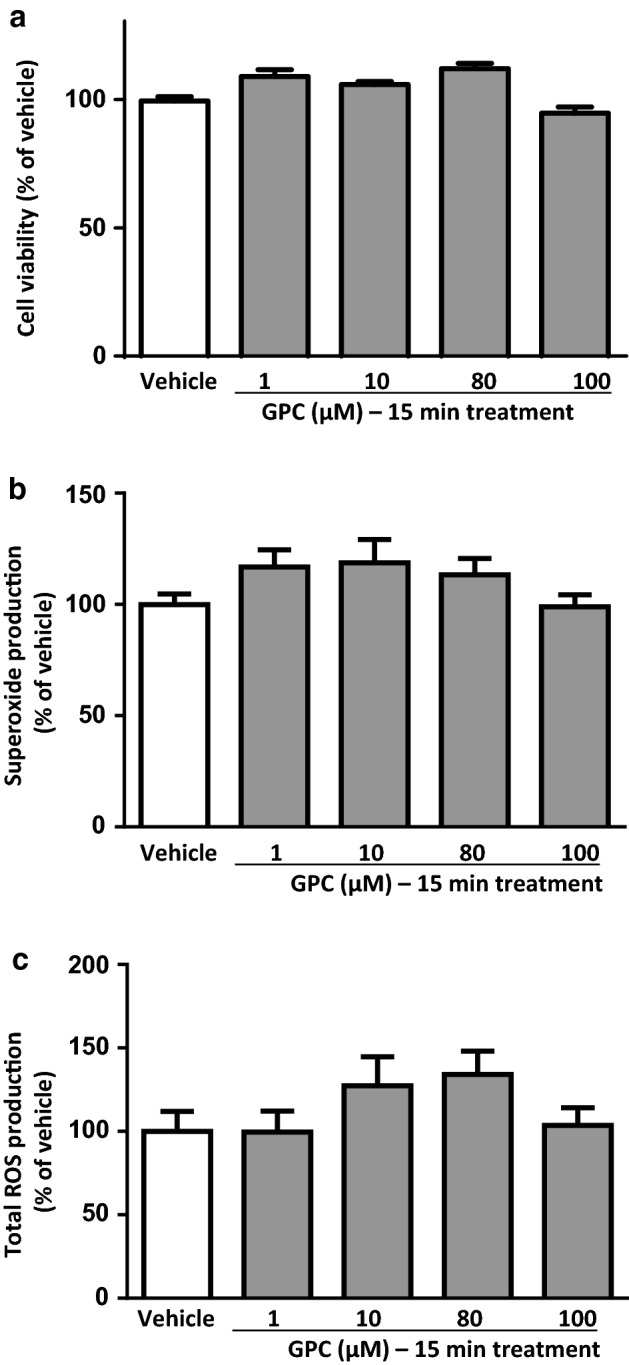
The acute effect of different concentrations of GPC on **a** cell viability **b** superoxide production and **c** total ROS production in isolated NRCMs after 15 min of GPC treatment are shown. Results are normalized to vehicle-treated cells. Data are presented as mean ± S.E.M. Statistical analysis of data was performed by one-way ANOVA, followed by Dunnett’s multiple comparison test,*p < 0.05 versus vehicle-treated cells (*n* = 8–16)

In normoxic conditions, the acute (15 min) treatment with different concentrations of GPC had no impact on the cell viability of NRCMs in comparison to the vehicle control (Fig. 2a). Neither the extent of superoxide production nor total ROS accumulation changed significantly after 15-min exposure to GCP in cardiac myocytes (Fig. 2b, c). On the basis of our earlier findings by Strifler and co-workers [12], acute effects of GPC on mitochondrial respiration were also investigated in this setup, and interestingly, treatment with 80–100 µM GPC heavily increased the oxygen consumption rate (OCR) of cardiac myocytes (Supplementary Fig. 2).

### Short-term effect of GPC in NRCMs in normoxic conditions

**Fig. 3:**
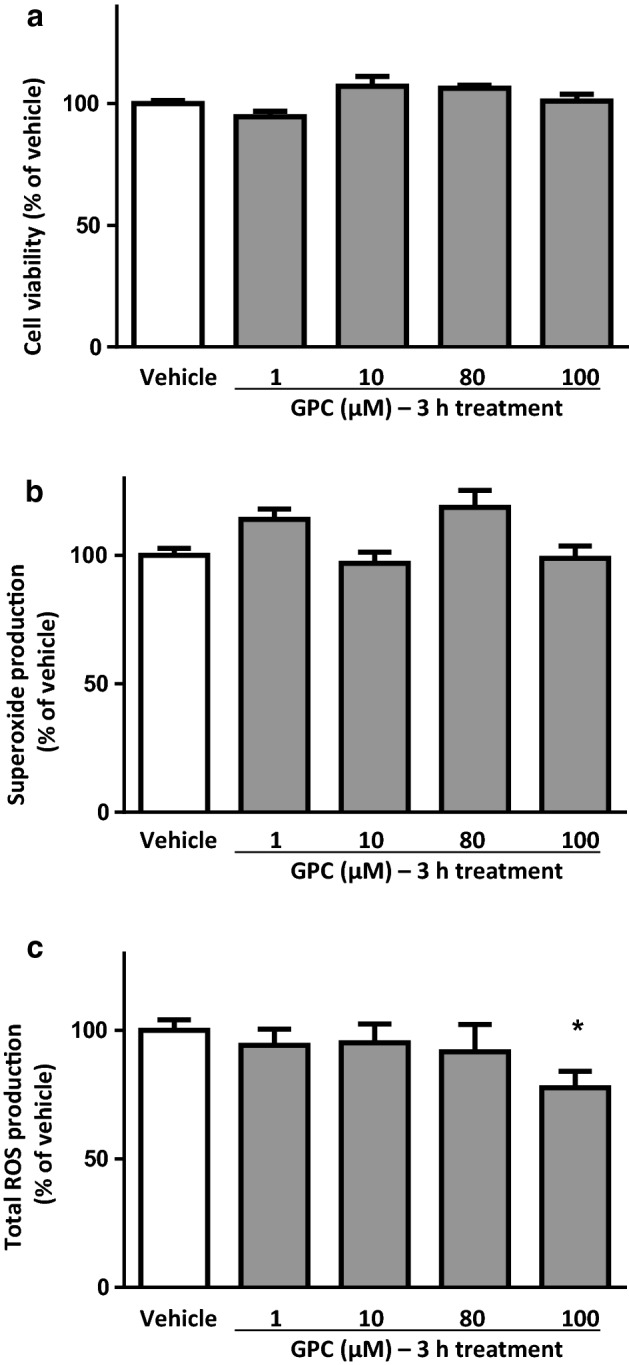
The short-term effect of different concentrations of GPC on **a** cell viability **b** superoxide production and **c** total ROS production in isolated NRCMs after 3 h of GPC treatment. Results are normalized to vehicle-treated cells. Data are presented as mean ± S.E.M. Statistical analysis of data was performed by one-way ANOVA, followed by Dunnett’s multiple comparison test,*p < 0.05 versus vehicle-treated cells (*n* = 8–16)

In normoxic conditions, similarly to those observations after acute administration, the short-term (3 h) GPC treatments did not change significantly the cell viability of NRCMs in comparison to the vehicle control (Fig. 3a).

In short-term applications of GPC, none of the applied concentrations had an influence on the superoxide level in cardiomyocytes (Fig. 3b). Nevertheless, a 3-h treatment with 100 µM GPC resulted in a significant reduction of the overall ROS production compared to the vehicle-treated group (Fig. 3c). Taken together this surprising observation with the above-mentioned OCR results, we suggest a direct intra-mitochondrial effect of exogenous GPC (as was noted in our previous study) between 80 and 100 µM concentration that was represented by a higher OCR and a subsequent physiological mitochondrial ROS formation. It was possibly followed by a general compensatory mechanism of the cells to scavenge the extended amount of ROS which was manifested in a significant decrease at 100 µM treatment.

### Effect of short-term application of GPC in isolated NRCMs exposed to SI/R

**Fig. 4:**
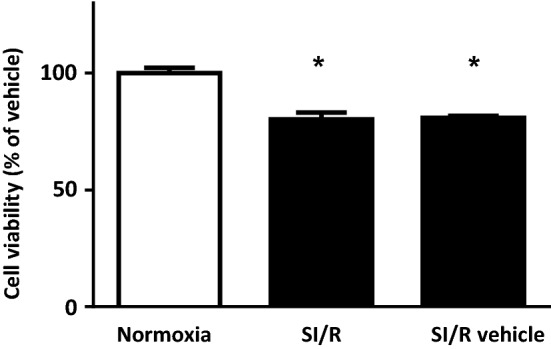
NRCMs which were encountered 4 h of simulated ischemia and 2 h reperfusion showed a significant decline in cell viability in comparison to the normoxic group. Data are presented as mean ± S.E.M. Statistical analysis of data was performed by one-way ANOVA, followed by Dunnett’s multiple comparison test,*p < 0.05 versus vehicle-treated cells (*n* = 8–16)

**Fig. 5:**
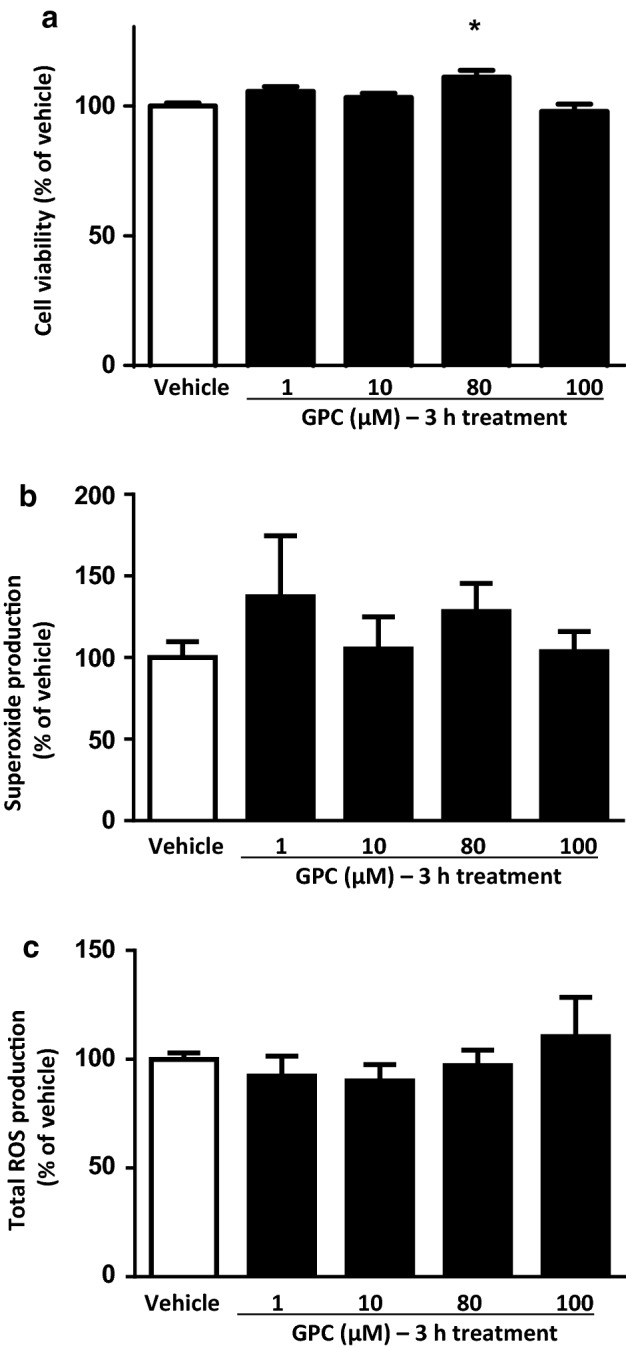
The effect of 3-h pre-treatment of GPC in different concentrations on NRCM’s **a** viability **b** superoxide production and **c** total ROS production after 4 h of simulated ischemia and 2-h reperfusion. Results are normalized to normoxic control group. Data are presented as mean ± S.E.M. Statistical analysis of data was performed by one-way ANOVA, followed by Dunnett’s multiple comparison test,*p < 0.05 versus normoxic controls, #*p* < 0.05 versus vehicle-treated cells (*n* = 8–16)

To validate our SI/R model, first, the viability of cells exposed to simulated ischemia (hypoxic solution combined with hypoxic environment) was compared with that observed after the normoxia protocol (normoxic solution combined with normoxic environment). Those NRCMs which were encountered to the described SI/R protocol and challenged with either hypoxic solution or vehicle showed a significant decline in cell viability in comparison to the normoxic group (Fig. 4a). Next, analysis of the degree of SI/R-caused reduction in cell viability was performed to compare the vehicle and the 3-h-long GPC pre-treated groups. Most of the applied concentrations of GPC had no effect on cell death. The 80 µM GPC pre-treatment, however, significantly improved cell viability after the SI/R challenge (Fig. 5a) and thereby rendered promising cytoprotective attribute.

In order to shed some lights on the antioxidant features of GPC in the chosen concentration range, superoxide production and the overall ROS formation of the cardiac cells were both investigated under SI/R conditions. Interestingly, none of the applied GPC concentrations did result in significant changes in these two parameters (Fig. 5b, c), which suggests us that the cytoprotective effect of GPC might be other than direct ROS-scavenger capacity.

### Effect of long-term GPC administration in isolated NRCMs in normoxic conditions

**Fig. 6:**
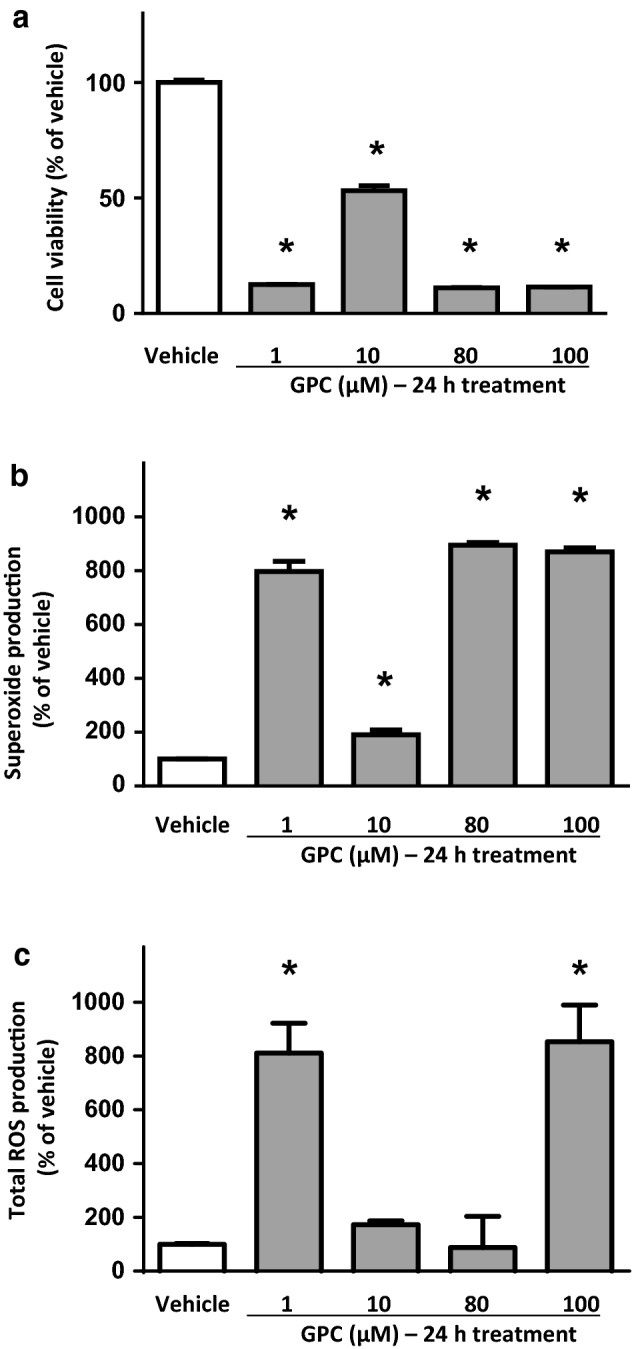
The long-term effect of different concentrations of GPC on **a** cell viability **b** superoxide production and **c** total ROS production in isolated NRCMs after 24 h of GPC treatment. Results are normalized to vehicle-treated cells. Data are presented as mean ± S.E.M. Statistical analysis of data was performed by one-way ANOVA, followed by Dunnett’s multiple comparison test,*p < 0.05 versus vehicle-treated cells (*n* = 8–16)

The long-term (24 h) GPC treatment of NRCMs induced profound cell death at each applied GPC concentration, as compared to the vehicle-treated group (Fig. 6a). In accordance with these findings, the intracellular superoxide levels significantly increased at each applied concentration of GPC (Fig. 6b). Additionally, the overall ROS concentrations significantly increased at 1 µM and 100 µM concentration of GPC (Fig. 6c) which might reflect some interesting crosstalk between mitochondrial and other intracellular ROS-scavenger mechanisms.

## Discussion

In our study, we sought to provide new pieces of evidence for the potential cardioprotective properties of GPC per se and under SI/R conditions. It has been increasingly recommended to use as a nutraceutical compound. Finding a safe and beneficial therapeutic window for its application is crucial. To date, no effects on cardiac tissue or cells of this compound are described. To confirm its biosafety, first, the normoxic experiments were carried out. Neither the acute, 15-min nor the short-term, 3-h exposure to GPC affected the cell viability of neonatal rat cardiac myocytes. Nevertheless, significant, acute effects of GPC at 80–100 µM concentration were noted as regards mitochondrial respiration which was considered as a part of some physiological intra-mitochondrial ROS-producing mechanism that was in line with the short-term ROS reduction at 100 µM treatment. It is proposed that the cells might harbor compensatory increases in other ROS-scavenging mechanisms which are switched on in response to elevated mitochondrial respiration.

Next, SI/R challenge was performed to ascertain some protective potential of GPC. A 3-h treatment with 80 µM concentration of GPC had a cytoprotective effect as indicated by the cell viability of cells challenged with SI/R. No significant changes were recorded regarding the pro-oxidant parameters although a strong propensity for an increase in the total ROS level but not the superoxide quantity was considered at 80 and 100 µM GPC concentration.

Regarding the normoxic, 24-h experiments, long-term exposure to each GPC concentration in normoxic conditions resulted in extensive cellular death of the cardiac myocytes alongside with significantly increased overall ROS level at each applied concentration. To the best of our knowledge, this is the first demonstration of the potential cardiotoxicity of GPC. We strongly recommend these findings to be taken into account and investigated in further details before designing clinical trials with chronic GPC administration in the future.

The most recent human study on high dosages of GPC examined the effects of a 6-day treatment on muscle strength [18], which showed limited effectiveness. According to the authors, there is a considerable issue with the current research on GPC because of the large variety in terms of dosage. Some studies examining GPC dosages up to 1000 mg (approx. 4 mM) per se [1] and others describe a dosage within a nutritional supplement range that was at a dose of 150 mg (approx. 600 µM) [19]. There is a huge variation in the literature regarding acute versus long-term administration. The majority of studies used dosages of around 600 mg (approx. 2.4 mM) in an acute fashion, although the chronic dosing may vary heavily based on the health condition of the human subjects and could reach a daily dosage of 1000 mg for 28 days [7].

There is broad, reliable evidence for GPC tolerance in laboratory animals. Acute oral, as well as parenteral toxicity of GPC, has been shown to be very low in several animal species including mice, rats, and dogs (LD_50_ > 1000 mg/kg i.v./i.p. in rodents; LD_50_ > 500 mg/kg i.m. and LD_50_ > 2000 mg/kg p.o. in dogs). Additionally, sub-chronic and chronic oral toxicity of GPC is considered to be very low. A 4-week administration of 100 and 300 mg/kg GPC in rats did not alter behavior nor produce any signs of general toxicity. A 26-week administration of 300 mg/kg GPC did not produce any toxic effects in rats and it showed only mildly reduced activity in dogs. A high dose of 1000 mg/kg induced reduced activity in rats [4]. In addition, GPC is considered not to be genotoxic both in vitro and in vivo: it exerts no mutagenicity in as high a dose as 3000 µg/mL of concentration in yeasts, and at 10.000 µg/plate concentration in bacterial colonies [4]. With respect to its toxicity, it should be pointed out that while GPC toxicity was tested mostly in vivo following oral administration [4], in the present study, we have tested the direct in vitro effects of GPC on neonatal rat cardiomyocyte cultures. Since GPC is hydrolyzed in the gut mucosa [2], in vivo (after oral administration) and in vitro effects of GPC may differ. Nevertheless, cardiac toxicity and cardiac effects of orally administered GPC have not yet been reported. Orally administered GPC showed several beneficial, non-cardiac effects such as neuroprotective effects in different types of brain damage [20–22] and mental disorders [23], as well as in acute stroke or transient ischemic attacks [7]. It has also been shown that GPC can rapidly cross the blood–brain barrier [11]. Applied GPC doses in these studies were relatively high. It is important in future in vivo experiments to examine the dose–response relationship between GPC and risk organs which are different from the target one, with a special emphasis on the possible side effects.

Firstly in our study, we assessed the impact of different exogenous GPC doses on healthy cardiac myocytes in normoxic conditions. Our chosen concentration range of GPC (1–100 µM) was based on pilot experiments and on papers describing the free choline concentrations in plasma [24, 25]. The viability of cells was not influenced by any of the applied GPC concentrations in any of the experimental setups, except for the 24 h treatment at which time point profound superoxide and overall ROS accumulation were also detected. Other in vitro studies indicated the emerging oxidative stress of 24-h GPC administration applied on astrocytes [26] and excessive neuronal cell death was described in another recent scenario in response to exogenous, chronic GPC [27]. It was also reported that in vitro, long-term exposure to muscarinic agonists significantly reduced the expression of a series of proteins belonging to the cytoskeletal structure and led to a complete change in the cellular shape. This possibly made the cells more prone to be disturbed by inflammatory factors [28]. In our case, this latter was likely due to the increasing amount of ROS. This was fully supported by our data. Indeed, after 24 h of GPC treatment, a significant increase in both the superoxide and overall ROS formation was observed at 1 and 100 µM, and superoxide level was detected to be significantly higher at 80 µM. In line with these results, cellular viability was significantly reduced at 1, 80, and 100 µM concentrations, respectively. GPC is previously reported to increase inositol phosphate (IP) formation and to promote PKC translocation in the brain [29]. However (to date), no similar observations were obtained in cardiac cells. In fact, it is well known that cardiac dysfunction with marked changes in the membrane inositol phosphate-Protein Kinase C (IP-PKC)-mediated signalization and subsequent Ca^2+^-handling abnormalities in cardiac myocytes arise primarily from oxidative stress as well as reduced antioxidant defenses [30]. It is thus possible that long-term exposure to a modulator of the IP-PKC cascade led to a critical accumulation of ROS with inescapable cell death. The possible explanation of the long-term cytotoxic effect might also be due to the feature of GPC as being a muscarinic acetylcholine receptor (mAChR) agonist [6]. Several mAChR subtypes may be potentially linked to the ROS production through multiple effector cascades, i.e., by elevating intracellular calcium levels, activating PKC, or by increasing the formation of arachidonic acid through phospholipase A2 activation [31]. More recently, long-term stimulation of ACh production by cigarette smoke extract for 4 days was reported to enhance the traditional PI3/PKC/PEBP1/Raf-ERK1/2 pathway activation as well as the release of pro-inflammatory and oxidative mediators in 16HBE cell line in vitro [32]. The existence of different subtypes of mAChRs on rat neonatal cardiomyocytes is well supported [33].

Additionally, an action of the 80 µM GPC was seen in the long-term scenario at which concentration a complete attenuation in the overall ROS formation but not in the superoxide release was detected. Theoretically, this might point out an interplay of GPC in physiological ROS formation and cardiac mitochondria. As previously mentioned, acute treatment with 80 and 100 µM GPC increased the oxygen consumption rate (OCR) of healthy cardiac myocytes and tendentiously increased the superoxide level. The total ROS staining results of the short-term scenario match this observation, since a significant reduction was seen at 100 µM GPC but regarding the superoxide concentrations, changes remained unnoticed. Physiological ROS formation and the redox signaling of cardiac cells through superoxide play an important role to maintain the normal function [34]. Additionally, 80–100 µM GPC could promote the physiological need of superoxide through a direct mitochondrial effect, which was then attenuated at the 100 µM GPC through some general ROS-scavenging compensatory mechanism as only the overall ROS level but not the superoxide quantity was decreased. This action was likely to be uncontrolled and therefore exaggerated in the case of the long-term GPC treatment, when the 100 µM dose was not enough to reverse the deleterious process.

Previous studies have shown that GPC is able to prevent oxidative stress and decrease radical production in different I/R models, e.g., liver or mesenteric tissue [12–14]. It has also been demonstrated that dietary supplementation with a mixture of GPC and other four compounds reduced the production of ROS in mice brain [35]. At this point in time, no myocardial protection effects were investigated related to GPC. Therefore, the same concentrations of GPC treatment as used in normoxic conditions were applied in a clinically relevant I/R model of cardiac myocytes. After SI/R protocol, viability assay was performed and showed that 80 µM GPC treatment resulted in a significant improvement of cell viability after SI/R. This observation suggested that GPC in 80 uM concentration was cytoprotective against I/R, likely via facilitating some as-of-yet un-described, fine-tuning mechanism in ROS-mediated cell signalization/cellular death pathways.

There are some limitations in the present study as this is the first study to investigate the effects of a broad concentration range of GPC on cardiac cells. Nevertheless, in vivo experiments would certainly supply further information regarding the functional cardiac effects of GPC. The mechanisms of action of GPC on cardiac cells are poorly characterized, and therefore it should be studied with multiple omics technologies in any depth.

In conclusion, in the present study, we have shown for the first time that choline donor l-Alpha-GPC (which is widely used as a food supplement and generally considered as safe for human use) had ambiguous effects on cardiac cells. It may be beneficial in short-term administration to maintain the physiological balance of ROS production under normoxic, healthy conditions and could be also protective in I/R conditions, but could, in fact, be cytotoxic if it surrounded the cells for long enough. Besides the duration of the treatment, the correct dosage can also be a crucial factor, as a fine-tuning effect seemed to occur in a small, but dietary-relevant concentration range. Thus (despite many limitations of this in vitro study), our results indicate the need for a comprehensive cardiac safety testing of GPC.

## Electronic supplementary material

Below is the link to the electronic supplementary material.

Supplementary material 1 (DOCX 195 kb)

## References

[1] Kawamura T, Okubo T, Sato K, Fujita S, Goto K, Hamaoka T, Iemitsu M (2012). Glycerophosphocholine enhances growth hormone secretion and fat oxidation in young adults. Nutrition.

[2] Abbiati G, Fossati T, Lachmann G, Bergamaschi M, Castiglioni C (1993). Absorption, tissue distribution and excretion of radiolabelled compounds in rats after administration of [14C]-l-Alpha-glycerylphosphorylcholine. Eur J Drug Metab Pharmacokinet.

[3] Lopez CM, Govoni S, Battaini F, Bergamaschi S, Longoni A, Giaroni C, Trabucchi M (1991). Effect of a new cognition enhancer, alpha-glycerylphosphorylcholine, on scopolamine-induced amnesia and brain acetylcholine. Pharmacol Biochem Behav.

[4] Brownawell AM, Carmines EL, Montesano F (2011). Safety assessment of AGPC as a food ingredient. Food Chem Toxicol.

[5] Zeisel SH, Da Costa KA, Franklin PD, Alexander EA, Lamont JT, Sheard NF, Beiser A (1991). Choline, an essential nutrient for humans. FASEB J.

[6] Muccioli G, Raso GM, Ghe C, Di Carlo R (1996). Effect of l-Alpha glycerylphosphorylcholine on muscarinic receptors and membrane microviscosity of aged rat brain. Prog Neuropsychopharmacol Biol Psychiatry.

[7] Barbagallo Sangiorgi G, Barbagallo M, Giordano M, Meli M, Panzarasa R (1994). alpha-Glycerophosphocholine in the mental recovery of cerebral ischemic attacks. An Italian multicenter clinical trial. Ann N Y Acad Sci.

[8] Moreno MDJM (2003). Cognitive improvement in mild to moderate Alzheimer’s dementia after treatment with the acetylcholine precursor choline alfoscerate: a multicenter, double-blind, randomized, placebo-controlled trial. Clin Ther.

[9] Parnetti L, Mignini F, Tomassoni D, Traini E, Amenta F (2007). Cholinergic precursors in the treatment of cognitive impairment of vascular origin: ineffective approaches or need for re-evaluation?. J Neurol Sci.

[10] Traini E, Bramanti V, Amenta F (2013). Choline alphoscerate (alpha-glyceryl-phosphoryl-choline) an old choline- containing phospholipid with a still interesting profile as cognition enhancing agent. Curr Alzheimer Res.

[11] Lee SH, Choi BY, Kim JH, Kho AR, Sohn M, Song HK, Choi HC, Suh SW (2017). Late treatment with choline alfoscerate (l-Alpha glycerylphosphorylcholine, alpha-GPC) increases hippocampal neurogenesis and provides protection against seizure-induced neuronal death and cognitive impairment. Brain Res.

[12] Strifler G, Tuboly E, Gorbe A, Boros M, Pecz D, Hartmann P (2016). Targeting mitochondrial dysfunction with L-alpha glycerylphosphorylcholine. PLoS ONE.

[13] Hartmann P, Fet N, Garab D, Szabo A, Kaszaki J, Srinivasan PK, Tolba RH, Boros M (2014). l-Alpha-glycerylphosphorylcholine reduces the microcirculatory dysfunction and nicotinamide adenine dinucleotide phosphate-oxidase type 4 induction after partial hepatic ischemia in rats. J Surg Res.

[14] Tokes T, Tuboly E, Varga G, Major L, Ghyczy M, Kaszaki J, Boros M (2015). Protective effects of l-Alpha-glycerylphosphorylcholine on ischaemia-reperfusion-induced inflammatory reactions. Eur J Nutr.

[15] Homocysteine Studies C (2002). Homocysteine and risk of ischemic heart disease and stroke: a meta-analysis. JAMA.

[16] Glunde K, Bhujwalla ZM, Ronen SM (2011). Choline metabolism in malignant transformation. Nat Rev Cancer.

[17] Gorbe A, Giricz Z, Szunyog A, Csont T, Burley DS, Baxter GF, Ferdinandy P (2010). Role of cGMP-PKG signaling in the protection of neonatal rat cardiac myocytes subjected to simulated ischemia/reoxygenation. Basic Res Cardiol.

[18] Bellar D, LeBlanc NR, Campbell B (2015). The effect of 6 days of alpha glycerylphosphorylcholine on isometric strength. J Int Soc Sports Nutr.

[19] Hoffman JR, Ratamess NA, Gonzalez A, Beller NA, Hoffman MW, Olson M, Purpura M, Jager R (2010). The effects of acute and prolonged CRAM supplementation on reaction time and subjective measures of focus and alertness in healthy college students. J Int Soc Sports Nutr.

[20] Tomassoni D, Avola R, Mignini F, Parnetti L, Amenta F (2006). Effect of treatment with choline alphoscerate on hippocampus microanatomy and glial reaction in spontaneously hypertensive rats. Brain Res.

[21] Ricci A, Bronzetti E, Vega JA, Amenta F (1992). Oral choline alfoscerate counteracts age-dependent loss of mossy fibres in the rat hippocampus. Mech Ageing Dev.

[22] Ciriaco E, Bronzetti E, Caporali MG, Germana GP, Niglio T, Piccolo G, Ricci A, Scotti De Carolis A, Amenta F (1992). Effect of choline alfoscerate treatment on changes in rat hippocampus mossy fibres induced by monolateral lesioning of the nucleus basalis magnocellularis. Arch Gerontol Geriatr.

[23] Sigala S, Imperato A, Rizzonelli P, Casolini P, Missale C, Spano P (1992). l-Alpha-glycerylphosphorylcholine antagonizes scopolamine-induced amnesia and enhances hippocampal cholinergic transmission in the rat. Eur J Pharmacol.

[24] Veenema K, Solis C, Li R, Wang W, Maletz CV, Abratte CM, Caudill MA (2008). Adequate Intake levels of choline are sufficient for preventing elevations in serum markers of liver dysfunction in Mexican American men but are not optimal for minimizing plasma total homocysteine increases after a methionine load. Am J Clin Nutr.

[25] Nurk E, Refsum H, Bjelland I, Drevon CA, Tell GS, Ueland PM, Vollset SE, Engedal K, Nygaard HA, Smith DA (2013). Plasma free choline, betaine and cognitive performance: the Hordaland Health Study. Br J Nutr.

[26] Grasso S, Bramanti V, Tomassoni D, Bronzi D, Malfa G, Traini E, Napoli M, Renis M, Amenta F, Avola R (2014). Effect of lipoic acid and alpha-glyceryl-phosphoryl-choline on astroglial cell proliferation and differentiation in primary culture. J Neurosci Res.

[27] de Pablo Y, Nilsson M, Pekna M, Pekny M (2013). Intermediate filaments are important for astrocyte response to oxidative stress induced by oxygen-glucose deprivation and reperfusion. Histochem Cell Biol.

[28] Stamatiou R, Paraskeva E, Vasilaki A, Mylonis I, Molyvdas PA, Gourgoulianis K, Hatziefthimiou A (2014). Long-term exposure to muscarinic agonists decreases expression of contractile proteins and responsiveness of rabbit tracheal smooth muscle cells. BMC Pulm Med.

[29] Aleppo G, Nicoletti F, Sortino MA, Casabona G, Scapagnini U, Canonico PL (1994). Chronic l-alpha-glyceryl-phosphoryl-choline increases inositol phosphate formation in brain slices and neuronal cultures. Pharmacol Toxicol.

[30] Tappia PS, Asemu G, Rodriguez-Leyva D (2010). Phospholipase C as a potential target for cardioprotection during oxidative stress. Can J Physiol Pharmacol.

[31] Naarala J, Tervo P, Loikkanen J, Savolainen K (1997). Cholinergic-induced production of reactive oxygen species in human neuroblastoma cells. Life Sci.

[32] Albano GD, Bonanno A, Moscato M, Anzalone G, Di Sano C, Riccobono L, Wenzel SE, Profita M (2018). Crosstalk between mAChRM3 and beta2AR, via acetylcholine PI3/PKC/PBEP1/Raf-1 MEK1/2/ERK1/2 pathway activation, in human bronchial epithelial cells after long-term cigarette smoke exposure. Life Sci.

[33] Yang CM, Chen FF, Sung TC, Hsu HF, Wu D (1993). Pharmacological characterization of muscarinic receptors in neonatal rat cardiomyocytes. Am J Physiol.

[34] Zhang M, Shah AM (2014). ROS signalling between endothelial cells and cardiac cells. Cardiovasc Res.

[35] Suchy J, Chan A, Shea TB (2009). Dietary supplementation with a combination of alpha-lipoic acid, acetyl-l-carnitine, glycerophosphocoline, docosahexaenoic acid, and phosphatidylserine reduces oxidative damage to murine brain and improves cognitive performance. Nutr Res.

