# Supplementary material for: l-Alpha-glycerylphosphorylcholine can be cytoprotective or cytotoxic in neonatal rat cardiac myocytes: a double-edged sword phenomenon
Source: Mol Cell Biochem. 2019 Jul 6;460(1):195–203. doi: 10.1007/s11010-019-03580-1 (PMC6745025; doi:10.1007/s11010-019-03580-1)
Supplement: Supplementary file 1 — Supplementary material 1 (DOCX 195 kb) [file 11010_2019_3580_MOESM1_ESM.docx]

Supplementary material

**Materials and methods**

**High resolution respirometry**

*In vitro* tests were performed using cardiac mitochondria and high resolution respirometry (HRR, (Oxygraph-2k high resolution respirometer, Oroboros Instruments, Innsbruck; Austria) to analyze the effects of GPC on mitochondrial respiration. During the measurements, mitochondria undergo different “states” by the sequential addition of substrates or inhibitors and the respiratory capacity could be assessed at multiple levels of the respiratory chain. Briefly, cardiac samples from newborn Wistar rats were homogenized in 1 ml of MitOx respiration medium (120 mM KCl, 20 mM HEPES, 10 mM KH_2_PO_4_, 86 mM MgCl_2_, 0,025% BSA) with a glass Potter homogenizer, and subsequently, 50 µl of homogenates were immediately placed into the detection chambers, which were calibrated to 200 nmol/ml oxygen concentrations in room air. The respirometry data were normalized to wet weight.

**Experimental protocol**

In this series, 1 µM - 100 µM GPC solutions were used in order to determine the effects of GPC on mitochondrial respiration (Supp. Fig. 2). First, the steady-state basal oxygen consumption of the homogenates (basal respiration) was measured. Then, the complex II-linked respiration (state II) was determined after the addition of 0.5 μM rotenone (complex I inhibitor) and 10 mM succinate (complex II substrate). Subsequently, the complex II-linked oxidative phosphorylation capacity (state III respiration) was estimated by adding saturating concentration of ADP to the medium.

**
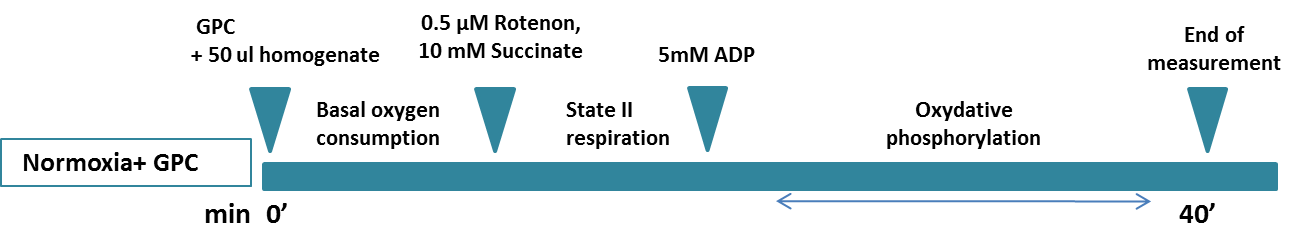
**

**Supplementary Figure 1.** Experimental protocol of oxidative phosphorylation measurement on neonatal cardiac mitochondria.

**Results**

The complex II-linked oxidative phosphorylation capacity of mitochondria changed significantly when 100 µM GPC was applied prior to respirometry as compared to the non-treated control (Supp. Fig. 2).


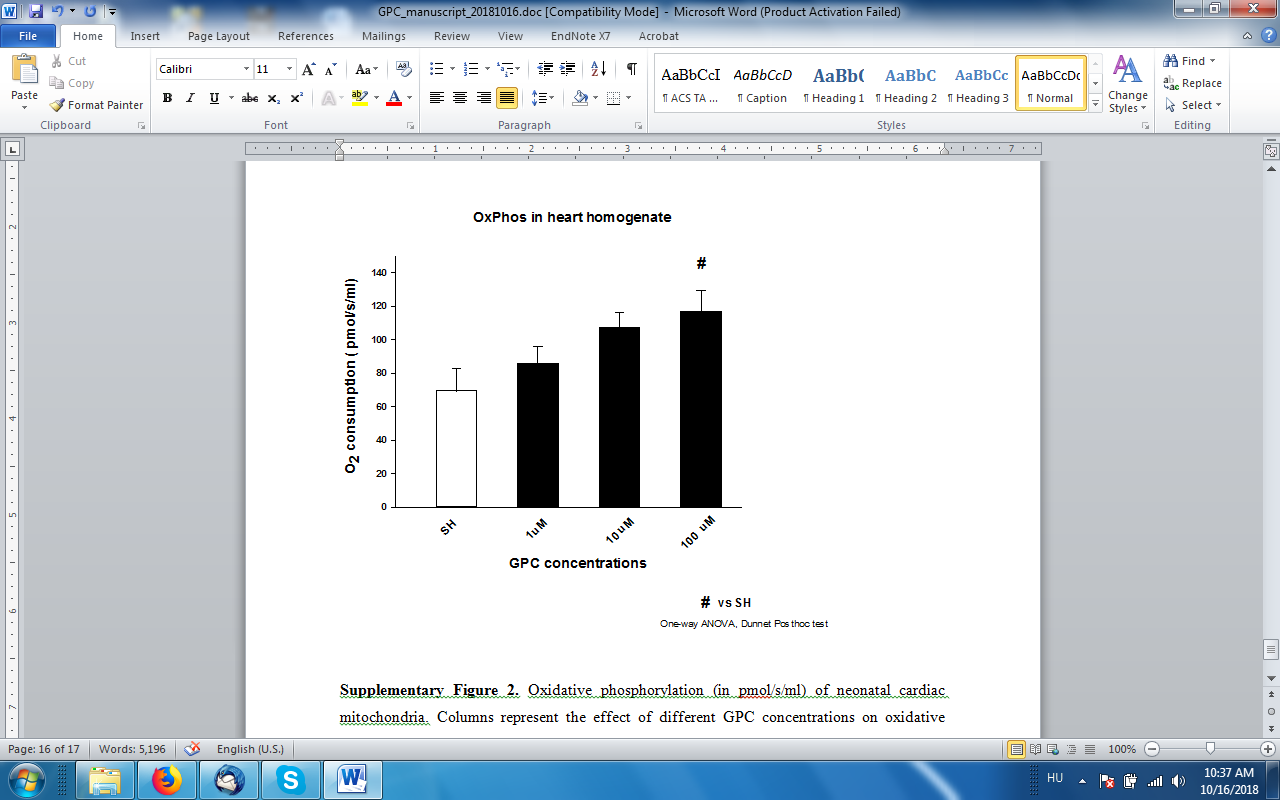


**Supplementary Figure 2.** Oxidative phosphorylation (in pmol/s/ml) of neonatal cardiac mitochondria. Columns represent the effect of different GPC concentrations on oxidative phosphorylation of cardiac mitochondria. Data are means ± SEM. Statistical analysis of data was performed by One-Way ANOVA, followed by Dunnett’s multiple comparison test, #p<0.05 vs SH.
